# Modification and validation of the Padua prediction score for assessing the risk of venous thromboembolism in medical inpatients: a case-control study

**DOI:** 10.1016/j.clinsp.2026.101071

**Published:** 2026-09-02

**Authors:** Shuhan Liu, Chunyu Feng, Yida Yu, Ruihuan Xu, Luling Ma, Ziwei Dai, Xiaohong Huang

**Affiliations:** a Department of Nursing (Hospital Infection Control Unit), Chongqing Red Cross Hospital (Jiangbei District People's Hospital), Chongqing, China; b Clinical Medical College of Shenzhen, Guangzhou University of Chinese Medicine, Shenzhen, Guangdong, China; c Department of General Surgery, Chongqing Red Cross Hospital (Jiangbei District People's Hospital), Chongqing, China; d Department of Laboratory Medicine, The Second Affiliated Hospital of The Chinese University of Hong Kong (Shenzhen) School of Medicine, Shenzhen, Guangdong, China; e Department of Nursing, Longgang District Central Hospital of Shenzhen, Shenzhen, Guangdong, China

**Keywords:** Venous thromboembolism, The Padua prediction score, Thromboprophylaxis, Medical inpatients, Case-control study

## Abstract

**Aim:**

To modify and validate the Padua prediction score in Chinese medical inpatients.

**Design:**

A case-control study.

**Methods:**

The clinical data of 663 Chinese internal medicine inpatients provided the basis for optimizing the model. Independent risk factors for Venous Thromboembolism (VTE) were analyzed by multifactorial logistic regression, and a modified risk assessment model was designed. Then 414 patients were selected as the external validation group, and the Receiver Operating Characteristic (ROC) and the McNemar test were used to assess the clinical applicability and discriminative power of the score.

**Results:**

Obesity (body mass index ≥ 25 kg/m^2^), varicose veins, diabetes mellitus, and D-dimer ≥0.5 µg/mL were independent risk factors for the development of VTE in Chinese internal medicine inpatients, with a corresponding risk score of 1. When the modified Padua risk assessment model took the best critical value, the Area Under the Curve (AUC) was 0.80, the accuracy was 0.75, the sensitivity was 0.82, the specificity was 0.69, the positive predictive value was 0.67, and the negative predictive value was 0.83. McNemar test showed that there was a difference in the predictive effect results of the two scores, and, except for specificity, the modified score's predictive effects were better than those before modification.

**Conclusion:**

The Padua score with the proposed modifications was more effective in predicting the risk of venous thromboembolism in hospitalized Chinese individuals.

**Limitation:**

This study was based a limited number of cases and only proposed minor adjustments to the established scoring system.

## Highlights

• Modified Padua score for VTE risk in Chinese inpatients: obesity (BMI ≥25), varicose veins, diabetes, D-dimer ≥0.5 µg/mL.
• Improved Padua model achieves higher AUC (0.80), sensitivity (0.82), and accuracy (0.75) compared to the original version.
• External validation confirms the modified score's superior performance in identifying high-risk VTE patients.
• Adjusted obesity criteria (BMI ≥25) align with Asian population standards, improving clinical relevance.
• Supports targeted thromboprophylaxis in Chinese medical inpatients, optimizing resource allocation.

## Introduction

Venous Thromboembolism (VTE), the third most common cardiovascular disease in the world, is characterized by high incidence, mortality, disability, and recurrence, with nearly 10 million new cases reported each year.^1^^,^^2^ and 60% of these morbidities are associated with hospitalization.^3^

VTE, as a common in-hospital complication, accounts for one-third of the total number of complication events and is also an important cause of preventable deaths, and surveys have shown that about three-quarters of VTE deaths are in-hospital acquired VTE, while one-third of them exhibit sudden fatal Pulmonary Thromboembolism (PTE).^4^ Epidemiological investigations indicate that in a large autopsy cohort study encompassing over 20,000 hospitalized patients, the detection rate of Venous Thromboembolism (VTE) was approximately 15%; the detection rate of Pulmonary Embolism (PTE) was approximately 25%; and the detection rate of concurrent VTE and PTE was approximately 10%.^5^^,^^6^ Once a patient develops VTE, even if it is cured, about one-third of the cases will recur within ten years, and recurrence is often accompanied by a higher mortality rate.^7^ the mortality rate of elderly patients with recurrent VTE is 20.5%, and the mortality rate of cancer patients with recurrent VTE is 88.1% within ten years.^8^ which not only seriously affects the quality of life of patients, but is also the main hospitals to generate vicious medical disputes events cause, and has become one of the important factors in the global burden of disease.^9^, ^10^, ^11^

The importance of VTE prevention has been emphasized in several global guidelines.^12^, ^13^, ^14^, ^15^, ^16^ and correct identification of thrombotic risk stratification using VTE risk assessment tools is the key to taking appropriate preventive measures for patients. The International VTE Epidemiology Study showed that among nearly 70,000 patients in 358 hospitals in 32 countries, 51.8% of hospitalized patients were at risk for VTE, and of all hospital-acquired VTE, about 75% of them were hospitalized in internal medicine, and 70%‒80% of fatal PTE cases occurred in internal medicine, so much so that hospitalization for internal medicine diseases is itself a risk factor for VTE, but only 40% of internal medicine patients received thromboprophylaxis, and the overall prophylaxis rate was only 13%‒64%.^17^, ^18^, ^19^ which suggests that internal medicine hospitalized patients, as a high-risk group for VTE, receive clinical treatment with thromboprophylaxis is not favorable.

The Padua prediction score is considered to be the most suitable risk assessment model for internal medicine inpatients because of its simplicity and sensitivity.^20^ and has been widely validated worldwide. However, the use of this model in China is mostly directly borrowed, and some problems have been found in clinical practice. Some studies have concluded that it does not include clinical risk factors for possible VTE and some entries are not suitable for Asian populations.^12^^,^^20^, ^21^ therefore, the Padua risk assessment model was improved and validated by combining data from 1077 cases in two tertiary hospitals, to provide a reference for the construction of a VTE risk assessment tool that is more suitable for Chinese internal medicine inpatients.

## Material and methods

### Design

We conducted a case-control study and a validation study with the aim of constructing a VTE risk assessment tool that better fits the characteristics of hospitalized patients in China. This study is reported by the checklist of items that should be included in case-control study reports in the Strengthening the Reporting of Observational Studies in Epidemiology (STROBE) guidelines.^22^

### Patients and selection criteria

Internal medicine inpatients attending Longgang Central Hospital from January 2017 to January 2023 were selected as the study subjects of the modified set, while patients hospitalized in the Second Hospital of the Chinese University of Hong Kong School of Medicine (Shenzhen) during the same period were selected as the study subjects of the validation set.

Inclusion criteria: 1) Age ≥18 years; 2) Hospitalization duration ≥72 hours; 3) Patients hospitalized for internal medicine diseases (including those in the intensive care unit). Exclusion criteria: 1) Patients with incomplete clinical data; 2) Patients with a definite diagnosis of VTE before or within 48 hours of hospitalization; 3) Patients received prophylactic anticoagulation; 4) Patients exhibiting symptoms of VTE.

The diagnostic criteria for VTE were based on imaging examinations, and according to the diagnostic criteria, a total of 663 patients were included in the modified set of this study, of which 221 were patients with VTE and 442 were patients without VTE. A total of 414 patients were included in the validation set, of whom 179 were VTE patients and 235 were non-VTE patients. In the study, there were no patient dropouts, withdrawals, or loss to follow-up.

### Data collection

Two researchers retrospectively collected data from the hospital information system on demographic, diagnostic, and treatment information, as well as metrics associated with the Padua Prediction Score, for both groups of patients: 1) Baseline demographic and clinical characteristics: gender, age, and Body Mass Index (BMI). 2) Clinical information: hypertension, diabetes mellitus, smoking, family history of VTE, coronary artery disease, mechanical ventilation, lower-extremity edema, blood transfusion, central venous catheterization, Acute Exacerbation of Chronic Obstructive Pulmonary Disease (AECOPD), chronic kidney disease, lower-extremity varicose veins, D-dimer, and history of Inflammatory Bowel Disease (IBD). 3) Padua prediction score features: active cancer, previous VTE, reduced mobility, already known thrombophilic condition, recent (≤1 month) trauma and/or surgery, elderly age, heart and/or respiratory failure, acute myocardial infarction or ischemic stroke, acute infection and/or rheumatologic disorder, obesity, ongoing hormonal treatment.

To avoid errors in the collection process, researchers were uniformly trained on the model entries and the requirements for filling them out, and possible disagreements in the collection process were discussed and analyzed, leading to a consensus.

### Statistical analysis

Epidata 3.1 was used to enter the relevant information, and SPSS 26.0 statistical software was used to analyze the data statistically. Normally distributed continuous information was expressed as mean ± standard deviation, and a *t*-test was used for comparison between groups. Non-normally distributed continuous information was expressed as median (P25, P75), and a non-parametric rank sum test was used for comparison between groups. Categorical data was expressed as frequency (n) and percentage (%), and the chi-square test was used for comparison between groups. The optimal cut-off value was selected according to the Youden index, and the sensitivity, specificity, Positive Predictive Value (PPV), and Negative Predictive Value (NPV) before and after the modification were calculated with VTE diagnosis as the gold standard, and the predictive efficacy was evaluated by combining with the area under the ROC curve, and the McNemar test was performed before and after the modification of the model, and the difference was considered to be statistically significant at p < 0.05.

### Ethics approval and consent to participate

This study was approved by the Ethics Committee of the Shenzhen Clinical Medical College of Guangzhou University of Traditional Chinese Medicine and approved by the Hospital Ethics Committee after informed consent-free review (approval number: ECPJ021) and reported by the STROBE statement.

## Results

### Characteristics of the clinical profile of patients in the modified set

A total of 663 patients were included in the modified set of the study, 221 in the VTE group and 442 in the non-VTE group. In the VTE group, 199 patients were diagnosed with DVT, 22 patients were diagnosed with PTE, and 7 patients with DVT had combined PTE.

A comparison of the basic characteristics of patients in the modified set is shown in Table 1, and the results showed that internal medicine inpatients aged ≥ 70 years and with BMI ≥ 25 kg/m^2^ were more likely to develop VTE, and the difference was statistically significant (p < 0.05).

**Table 1:** Comparison of basic characteristics of patients in the modified set.

Variable	VTE group (n = 221)	Non-VTE group (n = 442)	χ^2^	p
Gender			1.91	0.167
Male, n (%)	115 (52.0)	255 (57.7)		
Female, n (%)	106 (48.0)	187 (42.3)		
Age (years)			27.82	<0.001^a^
< 70, n (%)	147 (66.5)	373 (84.4)		
≥ 70, n (%)	74 (33.5)	69 (15.6)		
BMI (kg/m^2^)			11.84	<0.001^a^
< 25, n (%)	212 (95.9)	387 (87.6)		
≥ 25, n (%)	9 (4.1)	55 (12.4)		

a p < 0.001.

The comparison of the diagnostic and therapeutic data of the patients in the modified set is shown in Table 2, which shows statistically significant differences in terms of diabetes mellitus, mechanical ventilation, lower extremity edema, blood transfusion, central venous catheterization, lower-extremity varicose veins, and D-dimer ≥ 0.5 µg/mL (p < 0.05).

**Table 2:** Comparison of diagnostic and therapeutic information of patients in the modified set.

Variable	VTE group (n = 221)	Non-VTE group (n = 442)	χ^2^	p
**Hypertension**			1.51	0.219
Yes, n (%)	83 (37.6)	188 (42.5)		
No, n (%)	138 (62.4)	254 (57.5)		
**Diabetes mellitus**			14.41	<0.001^b^
Yes, n (%)	47 (21.3)	46 (10.4)		
No, n (%)	174 (78.7)	396 (89.6)		
**Smoking**			0.19	0.663
Yes, n (%)	23 (10.4)	51 (11.5)		
No, n (%)	198 (89.6)	391 (88.5)		
**Family history of VTE**			‒	0.157
Yes, n (%)	1 (0.5)	0 (0)		
No, n (%)	220 (99.5)	442 (100.00)		
**Coronary artery disease**			1.98	0.159
Yes, n (%)	30 (13.6)	79 (17.9)		
No, n (%)	191 (86.4)	363 (82.1)		
**Mechanical ventilation**			4.68	0.031^a^
Yes, n (%)	8 (3.6)	4 (0.9)		
No, n (%)	213 (99.4)	438 (99.1)		
**Lower-extremity edema**			15.47	<0.001^b^
Yes, n (%)	35 (15.8)	28 (6.3)		
No, n (%)	186 (84.2)	414 (93.7)		
**Blood transfusion**			5.28	0.022^a^
Yes, n (%)	28 (12.7)	32 (7.2)		
No, n (%)	193 (87.3)	410 (72.8)		
**Central venous catheterization**			9.43	0.002^a^
Yes, n (%)	20 (9.1)	15 (3.4)		
No, n (%)	201 (90.9)	427 (96.6)		
**AECOPD**			0.07	0.795
Yes, n (%)	11 (5.0)	20 (4.5)		
No, n (%)	210 (95.0)	422 (95.5)		
**Chronic kidney disease**			0.02	0.885
Yes, n (%)	8 (3.6)	17 (3.9)		
No, n (%)	213 (96.4)	425 (96.1)		
**Lower-extremity varicose veins**			11.05	<0.001^b^
Yes, n (%)	11 (5.0)	4 (0.90)		
No, n (%)	210 (95.0)	438 (99.10)		
**D-dimer (ug/mL)**			151.85	<0.001^b^
< 0.5	24 (10.9)	271 (61.3)		
≥ 0.5	197 (89.1)	171 (38.7)		
**History of IBD**			-	1.000
Yes, n (%)	2 (0.90)	3 (0.7)		
No, n (%)	219 (99.10)	439 (99.3)		

a p < 0.05

b p < 0.001.

### Modification of the construction of the Padua prediction score

Independent risk factors for the occurrence of VTE in internal medicine inpatients were analyzed using multifactorial logistic regression, with the occurrence of VTE events in patients as the dependent variable, and the statistically significant risk factors in Tables 1, 2 as the independent variables. The results showed that varicose veins of the lower extremities.^23^ BMI ≥25 kg/m^2^, diabetes mellitus.^24^ and D-dimer ≥ 0.5 µg/mL were independent risk factors (p < 0.05), see Table 3. Combined with the literature study, the analysis summarized the two unreasonable entries (existing thrombosis tendency and obesity) in the Padua prediction score.^25^ in the domestic clinical application and carried out the substitution and adjustment, so as to form the modified Padua prediction score (Table 4).

**Table 3:** Multifactorial logistic regression analysis of VTE risk factors.

Factors	*B*	S.E	Wals	df	P	OR	95%CI
BMI (kg/m^2^)	0.91	0.28	11.04	1	<0.001^b^	2.49	1.45‒4.27
Diabetes mellitus	0.63	0.29	4.76	1	0.029^a^	1.87	1.07‒3.28
Lower-extremity varicose veins	1.85	0.73	6.51	1	0.011^a^	6.36	1.54‒26.33
D-dimer (ug/mL)	2.10	0.26	63.87	1	<0.001^b^	8.13	4.87‒13.60

a p < 0.05

b p < 0.001.

**Table 4:** Padua prediction score and modified Padua prediction score.

Padua prediction score^23^		Modified Padua prediction score	
Active cancer^a^	**3**	Active cancer^a^	**3**
Previous VTE^b^	**3**	Previous VTE^b^	**3**
Reduced mobility^c^	**3**	Reduced mobility^c^	**3**
Already known thrombophilic condition^d^	**3**	Recent (≤1 month) trauma and/or surgery	**2**
Recent (≤1 month) trauma and/or surgery	**2**	Lower-extremity varicose veins	**1**
Elderly age (≥70 years)	**1**	D-dimer ≥0.5 µg/mL	**1**
Heart and/or respiratory failure	**1**	Diabetes mellitus	**1**
Acute myocardial infarction or ischemic stroke	**1**	Elderly age (≥70 years)	**1**
Acute infection and/or rheumatologic disorder	**1**	Heart and/or respiratory failure	**1**
Obesity (BMI ≥30)	**1**	Acute myocardial infarction or ischemic stroke	**1**
Ongoing hormonal treatment	**1**	Acute infection and/or rheumatologic disorder	**1**
		Obesity (BMI ≥25)	**1**
		Ongoing hormonal treatment	**1**

a Patients with local or distant metastases and/or in whom chemotherapy or radiotherapy had been performed in the previous 6-months.

b Patients with the exclusion of superficial vein thrombosis.

c Bedrest with bathroom privileges (either due to patient’s limitations or on physicians order) for at least 3-days.

d Carriage of defects of antithrombin, protein C or S, factor V Leiden, G20210A prothrombin mutation, antiphospholipid syndrome.

### Characteristics of the clinical profile of patients in the validation set

A total of 414 patients were included in the validation set of this study, 179 in the VTE group and 235 in the non-VTE group. In the VTE group, 170 patients were diagnosed with DVT and 9 patients were diagnosed with PTE.

Among the assessment indicators included before and after the modification of the Padua model in the two groups of patients in the validation set, VTE and non-VTE groups, there were statistically significant differences in elderly age, ongoing hormonal treatment, recent (≤ 1-month) trauma and/or surgery, acute infection and/or rheumatologic disorder, active cancer, previous VTE, obesity, lower-extremity varicose veins, and D-dimer, as shown in Table 5.

**Table 5:** Comparison of entries in the validation set before and after score modification.

Variable	VTE group (n = 179)	Non-VTE group (n = 235)	χ^2^	p
**Gender**			0.33	0.566
Male, n (%)	97 (54.2)	134 (57.0)		
Female, n (%)	82 (45.8)	101 (43.0)		
**Elderly age (years)**			26.22	<0.001^e^
< 70, n (%)	84 (46.9)	54(23.0)		
≥ 70, n (%)	95 (53.1)	181(77.0)		
**BMI (kg/m^2^)**^a^			26.72	<0.001^e^
≥ 30, n (%)	4 (2.2)	9 (3.8)		
≥ 25, n (%)	119 (66.5)	97 (41.3)		
< 25, n (%)	60 (33.5)	138 (58.7)		
**Active cancer**			7.58	0.006^d^
Yes, n (%)	6 (3.4)	24 (10.2)		
No, n (%)	173 (96.6)	211 (89.8)		
**Previous VTE**			5.30	0.021^d^
Yes, n (%)	4 (2.2)	0 (0.0)		
No, n (%)	175 (97.8)	235 (100.0)		
**Reduced mobility**			0.58	0.447
Yes, n (%)	3 (1.7)	2 (0.9)		
No, n (%)	176 (98.3)	233 (99.1)		
**Already known thrombophilic conditionb**^b^			‒	‒
Yes, n (%)	0 (0.0)	0 (0.0)		
No, n (%)	179 (100.0)	235 (100.0)		
**Recent (≤ 1 month) trauma and/or surgery**			23.11	<0.001^e^
Yes, n (%)	43 (24.0)	17 (7.2)		
No, n (%)	136 (76.0)	218 (92.8)		
**Heart and/or respiratory failure**			0.13	0.716
Yes, n (%)	20 (11.2)	29 (12.3)		
No, n (%)	159 (88.8)	206 (81.7)		
**Acute myocardial infarction or ischemic stroke**			1.44	0.230
Yes, n (%)	38 (21.2)	39 (16.6)		
No, n (%)	141 (78.8)	196 (83.4)		
**Acute infection and/or rheumatologic disorder**			8.17	0.004^d^
Yes, n (%)	100 (55.9)	98 (41.7)		
No, n (%)	79 (44.1)	137 (58.3)		
**Ongoing hormonal treatment**			6.64	0.010^d^
Yes, n (%)	40 (22.3)	30 (12.8)		
No, n (%)	139 (77.7)	205 (87.2)		
**Lower-extremity varicose veins**^c^			90.55	<0.001^e^
Yes, n (%)	86 (48.0)	17 (7.2)		
No, n (%)	93 (52.0)	218 (92.8)		
**D-dimer (µg/mL)**^c^			27.94	<0.001^e^
Yes, n (%)	128 (71.5)	107 (45.5)		
No, n (%)	51 (28.5)	128 (54.5)		
**Diabetes mellitus**^c^			5.33	0.021^d^
Yes, n (%)	92 (51.4)	94 (40.0)		
No, n (%)	87 (48.6)	141 (60.0)		

a This variable has been adjusted.

b This variable has been replaced by a newly added variable.

c This variable is a newly added variable.

d p < 0.05

e p < 0.001.

### Validation of the modified Padua prediction score

The sensitivity and specificity of the modified Padua prediction score at different scores were analyzed by the software to determine its optimal cutoff value, and when the total assessment score was 3.5, the sensitivity and specificity were 0.82 and 0.69, respectively, and the Youden index was taken to a maximum value of 0.51, as shown in Table 6. Therefore, the optimal cutoff value of the modified Padua model was 3.5, that is, when the total assessment score was ≥ 4, then the patient is at high risk for VTE.

**Table 6:** Predictive efficacy of modified Padua score taking different cutoff values.

Cut-off value	Sensitivity	Specificity	Youden index
0.5	1	0.06	0.06
1.5	0.966	0.26	0.226
2.5	0.905	0.515	0.42
3.5	0.816	0.694	0.51
4.5	0.626	0.821	0.447
5.5	0.453	0.885	0.338
6.5	0.229	0.932	0.161
7.5	0.117	0.979	0.096
8.5	0.056	0.987	0.043
9.5	0.028	0.991	0.019
10.5	0.017	0.996	0.013
11.5	0.006	1	0.006
13	0	1	0

The patients in the validation set were assessed using the Padua risk model, and 71 high-risk patients and 343 low-risk patients were screened for possible VTE; 218 high-risk patients and 196 low-risk patients were screened for possible VTE using the modified Padua model. The 414 samples were subjected to paired crossover analysis, where 0 indicated the low-risk group of developing VTE and 1 indicated the high-risk group of developing VTE, and the results are shown in Table 7. The high-risk and low-risk groups predicted to be the same by the model were shown to be 71 cases and 196 cases, respectively, and McNemar's test showed that the predictive efficacy of the two models yielded inconsistent results, and the difference was statistically significant (χ^2^ = 147, *df* = 1, p < 0.001).

**Table 7:** Prediction results before and after Padua prediction score modification.

Padua Prediction Score	Modified Padua Prediction Score		Total
	0	1	
0	196	147	196
1	0	71	218
Total	343	71	414

According to the optimal cut-off value determined at the time of model development, the Padua score predicted VTE patients with an accuracy of 0.59, a sensitivity of 0.23, a specificity of 0.87, a PPV of 0.58, and an NPV of 0.60; the modified Padua score predicted VTE patients with an accuracy of 0.75, a sensitivity of 0.82, a specificity of 0.69, the PPV of 0.67, and NPV of 0.83, as shown in Table 8, and the AUCs before and after the modification were 0.74 and 0.80, respectively, which showed that the predictive ability of the modified Padua score was superior to that of the Padua score.

**Table 8:** Comparison of predictive efficacy before and after modification of Padua score.

	Cut-off	Youden index	AUC	p	Sensitivity	Specificity	PPV	NPV
**Before**	4	0.10	0.745	<0.001^a^	0.23	0.87	0.58	0.60
**After**	4	0.51	0.801	<0.001^a^	0.82	0.69	0.67	0.83

a p < 0.001.

## Discussion

VTE in hospitals is characterized by high incidence, high disability, and high mortality, posing a great threat to medical safety and quality, and has become a severe test for clinical workers and hospital administrators. The standardized prevention of VTE has become a key task of China's national healthcare quality and safety improvement for the fifth consecutive year. However, thromboprophylaxis for internal medicine patients has not yet been popularized, and there is a lack of effective risk assessment tools. Although the Padua prediction score is recommended by many guidelines.^16^^,^^26^, ^27^ and is the most widely used VTE risk model in internal medicine, the score is based on European populations and differs from the characteristics of Chinese internal medicine inpatients, which suggests that it is necessary to establish a VTE risk assessment standard that meets the characteristics of the Chinese population.

### Implications for policy and practice

In this study, we found that some of the entries in the original prediction scores did not apply to Asian people. Regarding the obesity standard, WHO has set different BMI standards for different regions, and BMI ≥30 in the original model is more suitable for European and American populations, whereas in Asia.^28^, ^29^ BMI ≥ 25 is considered obese, and reviewing the studies on the Padua Scale in China in recent years.^30^^,^^31^ the inclusion of BMI ≥ 30 in the subjects of the patients was mostly the number of individual cases, and there were only 4 cases (2.2%) in this study, so the definition of obesity in the Padua model was adjusted to BMI ≥ 25 by combining the criteria of obesity in China and Asia, which was consistent with the results of a Korean study.^32^ Among the entries of thrombosis tendency, the mutations of V Leiden factor and G20210A prothrombinogen are closely related to race and geography.^33^^,^^34^ which are more common in European and American populations, while the probability is extremely low in Asia, and the possibility of its being a major risk factor for VTE in Asian populations is extremely small, and several studies in China have also shown that the detection rate tends to be close to 0.^35^, ^36^, ^37^ which is in line with the present study, and This is consistent with the present study, and this type of test is not a routine program in hospitals, and the entry is easily voided in the process of modeling. Based on the analysis of the modified set data, it was concluded that obesity (BMI ≥ 25), varicose veins, D-dimer ≥ 0.5 µg/mL, and diabetes mellitus were the risk factors for internal medicine inpatients in China, which was consistent with the results of several studies.^38^, ^39^ which suggests that further attention should be paid to these risk factors in the future clinical work. Moreover, the results of the study showed that the improved score has better predictive efficacy, which can guide the clinic to accurately identify high-risk patients, help healthcare institutions carry out targeted preventive measures, reduce the occurrence of VTE events, and optimize the allocation of healthcare resources.

### Recommendations for further research

This study confirmed that the modified Padua model has better predictive efficacy in the assessment of VTE risk in internal medicine inpatients, but there are many factors affecting thrombosis, and the etiological composition of the enrolled patients has not been explored in depth, and different systems of internal medicine diseases may have a certain impact on the results of the study, which needs to be further investigated in depth. The local optimization of the improved Padua model should not be limited to the improvement of prediction performance, but the ultimate value is to reduce the incidence of VTE. However, no study has been carried out on the evaluation of the intervention effect, and a series of empirical studies are needed to prove its clinical value in order to promote individualized prevention.

### Strength and limitations of the work

In this study, we started with the localization of the risk prediction score, clarified the inapplicable items in the original score through literature research, and improved the original score based on the statistical analysis of the risk factors that better fit the characteristics of Chinese patients' medical records. The results showed that the improved score was not only highly operable but also significantly enhanced the ability to identify the risk of VTE in Chinese patients with internal medicine, which provided a practical tool for clinical screening. However, this study is limited by time and conditions, the sample size is small, and the included cases only came from two hospitals, so the sample is not representative enough, which has some limitations.

## Conclusions

This study modified and validated the Padua prediction score for Chinese medical inpatients. The modified model adjusted the obesity criterion to BMI ≥25 kg/m^2^ and incorporated lower extremity varicose veins, diabetes, and D-dimer ≥0.5 µg/mL as independent risk factors. Its predictive performance significantly surpassed the original score. This score better aligns with the characteristics of the Chinese population, aiding in the clinical identification of patients at high risk for venous thromboembolism and supporting the development of targeted preventive measures. Future multicenter prospective studies are still needed to further validate its applicability.

## CRediT Author Statement

Shuhan Liu (First Author): Conceptualization, Formal analysis, Investigation, Methodology, Visualization, Writing-original draft, Writing-review & editing.

Chunyu Feng: Data curation, Writing-original draft.

Yida Yu: Data curation, Investigation, Resources.

Ruihuan Xu: Resources, Supervision, Validation.

Luling Ma: Investigation, Resources.

Ziwei Dai: Investigation, Resources.

Xiaohong Huang (Corresponding Author): Conceptualization, Funding acquisition, Project administration, Supervision, Writing-review & editing.

Shuhan Liu and Chunyu Feng are co-first authors; they have contributed equally to this work and their contributions are the same. All authors have read and agreed to the published version of the manuscript.

## Data availability statement

The data supporting the results of this study are available from the corresponding author upon request. Relevant data are not publicly available due to privacy or ethical constraints.

## Declaration of competing interest

The authors declare no conflicts of interest.

## Appendix Supplementary materials

Image, application 1
